# Estimating heritability of glycaemic response to metformin using nationwide electronic health records and population-sized pedigree

**DOI:** 10.1038/s43856-021-00058-4

**Published:** 2021-12-01

**Authors:** Iris N. Kalka, Amir Gavrieli, Smadar Shilo, Hagai Rossman, Nitzan Shalom Artzi, Nancy-Sarah Yacovzada, Eran Segal

**Affiliations:** 1 Department of Computer Science and Applied Mathematics, Weizmann Institute of Science, Rehovot, Israel; 2 Department of Molecular Cell Biology, Weizmann Institute of Science, Rehovot, Israel; 3 Pediatric Diabetes Unit, Ruth Rappaport Children’s Hospital, Rambam Healthcare Campus, Haifa, Israel; 4 Department of Molecular Genetics, Weizmann Institute of Science, Rehovot, Israel

**Keywords:** Heritable quantitative trait, Type 2 diabetes

## Abstract

**Background:**

Variability of response to medication is a well-known phenomenon, determined by both environmental and genetic factors. Understanding the heritable component of the response to medication is of great interest but challenging due to several reasons, including small study cohorts and computational limitations.

**Methods:**

Here, we study the heritability of variation in the glycaemic response to metformin, first-line therapeutic agent for type 2 diabetes (T2D), by leveraging 18 years of electronic health records (EHR) data from Israel’s largest healthcare service provider, consisting of over five million patients of diverse ethnicities and socio-economic background. Our cohort consists of 80,788 T2D patients treated with metformin, with an accumulated number of 1,611,591 HbA1C measurements and 4,581,097 metformin prescriptions. We estimate the explained variance of glycated hemoglobin (HbA1c%) reduction due to inheritance by constructing a six-generation population-size pedigree from national registries and linking it to medical health records.

**Results:**

Using Linear Mixed Model-based framework, a common-practice method for heritability estimation, we calculate a heritability measure of ${h}^{2}=12.6 \%$ (95% CI, $6.1 \%\! -\!19.1 \%$) for absolute reduction of HbA1c% after metformin treatment in the entire cohort, ${h}^{2}=21.0 \%$ (95% CI, $7.8 \%\! -\!34.4 \%$) for males and ${h}^{2}=22.9 \%$ (95% CI, $10.0 \%\! -\!35.7 \%$) in females. Results remain unchanged after adjusting for pre-treatment HbA1c%, and in proportional reduction of HbA1c%.

**Conclusions:**

To the best of our knowledge, our work is the first to estimate heritability of drug response using solely EHR data combining a pedigree-based kinship matrix. We demonstrate that while response to metformin treatment has a heritable component, most of the variation is likely due to other factors, further motivating non-genetic analyses aimed at unraveling metformin’s action mechanism.

## Plain language summary

Individuals in a population might respond differently to the same medication and this phenomenon is commonly attributed to either genes or the environment. Here, we studied the familial aspects of the response to metformin, a medication used in the treatment of type 2 diabetes. We combined information from 18 years of medical records identifying newly treated patients with type 2 diabetes with information about how the trait was inherited within their families. We calculated a metric that tells us how well differences in people’s genes account for differences in their traits, and demonstrate that although the difference in response to metformin is in part explained by the genes people with type 2 diabetes inherit, most of it is not explained by genes. This finding contributes to a better understanding of differences in metformin response and might help inform treatment in future.

## Abstract (web summary)

Kalka and Gavrieli et al. assessed the heritability of variation in the glycaemic response to metformin by leveraging electronic health records data gathered from a large cohort of patients with diabetes and combining it with pedigree information. The authors show that although the variability in this response has a heritable component, most of it is likely non-genetic.

## Introduction

During the past three decades, there has been a twofold increase diabetes prevalence in the general population (WHO), currently estimated to afflict one in every 16 adults^1^. Type 2 diabetes (T2D), which accounts for ~90% of the total diabetic population, is a major cause of morbidity and is among the top ten mortality causes in adults^2,3^.

Metformin is the first-line oral agent for lowering blood sugar levels in T2D patients. Through inhibition of hepatic glucose production it reduces intestinal glucose absorption, and improves both glucose uptake and its utilization^4^. The significant role of metformin in T2D management is particularly remarkable since its mechanism is still not fully understood^5–7^.

Glycaemic response to metformin is varied across patients^6,7^, and remains unexplained by individual features. Some variation can be accounted for by personal characteristics including sex, age, and BMI, as well as features describing treatment strategies such as dosage and adherence^8^. In addition, a small fraction of the response variability is attributed to genetic variants, providing motivation to further explore heritable variance in metformin response^9^.

Medication response variations are widely agreed upon to be determined by the interplay of environmental and genetic factors^10,11^. The effect of heritable factors has been suggested as early as 1908^12^. This notion led to the development of pharmacogenomics, which investigates genetic variants that account for differential drug responses and personal responses to treatments^13^.

Traditionally heritability estimates are deciphered through twins and family studies, however, those are difficult to construct in the context of medication response. Drug response data, same diagnosis, and similar treatment are rarely available in multiple family members^14,15^. Moreover, because close relatives often share environment and not only genetics, such studies have difficulties in separating the genetic and environmental effects.

Other types of studies estimating the effect of genetic variability in drug responses rely on small cohorts undergoing costly genetic tests and use genetic relatedness estimation methods^16–19^. Some of these studies employ methods such as genome-wide complex trait analysis, which requires a large cohort, ideally greater than 10,000, however, most such cohorts are limited in their size resulting in estimates with low statistical power and do not represent the true distribution of the population^20,21^.

In studies bypassing genetic tests, such as family-linkage studies, information is highly sparse, and determining the response to medication by genetic and environmental factors is computationally challenging. Epigenetics may also play a role in the response to medication making the task even harder^22^.

Metformin’s effect is routinely measured through glycaemic control assessments using either fasting glucose or HbA1c%^23^. The latter is an indicator of blood sugar levels over the course of three months^24^, making it more reliable than the former, which is a snapshot of a single time point. Moreover, fasting glucose is affected by the strictness of fasting prior to the blood tests, an unrecorded measure, making fasting glucose more prone to mistakes.

In this study, we used Electronic Health Records (EHRs) from the Clalit Healthcare database, Israel’s largest healthcare service provider^25^. This population-size EHR provides a real-world view of the internal variability in healthcare systems, where patients, diagnoses, and treatment plans vary considerably. In general, EHRs can contain medical information on millions of patients, however, data are sparse and noisy, and not cross-sectional^26^. Combined with pedigree information from Israel’s national registries this unique data allowed us to include the family medical history of first-order relatives and extended family members alike.

Today, heritability estimation is typically performed using genotyping-based methods such as LD Score Regression from GWAS results^27^. Such models consider a matrix of standardized genotypes, estimating the heritability from the effects of the genetic variates that are accounted for. An alternative method was presented using Sparse Cholesky Factorization (Sci-LMM) package^28^, a statistical modeling framework for analyzing population-size pedigrees. Sci-LMM replaces the genetic matrix in a Linear Mixed Model (LMMs)^29^ with a vector that is sampled from the normal multi-dimensional distribution whose covariance matrix is a kinship matrix. The kinship matrix, commonly computed from genetic information, can be constructed from pedigree relationships solely from EHR information, without costly genetic testing. We estimated the heritability of absolute HbA1c% reduction in response to metformin to be ${h}^{2}=12.6 \%$ (95% CI, 6.1–19.1%) for the entire cohort, ${h}^{2}=21.0 \%$ (95% CI, 7.8–34.4%) for males and ${h}^{2}=22.9 \%$ (95% CI, 10.0–35.7%) in females of the total explained variability.

## Methods

### Data

We used EHRs of Clalit Health Services (Clalit), Israel’s largest healthcare provider. Clalit’s data are heterogeneous in terms of geography and socioeconomics, including more than five million people (over half of Israel’s population) with longitudinal measurements dating back to 2002. EHRs are reflective of the members’ full clinical experience including diagnoses, lab test results, and medication prescribed and dispensed. Patients’ information is combined with national registries to provide demographics consisting of the date of birth, sex, parental information, and county of birth, from which ethnicity is inferred^30^. The full-study protocol was approved by the Clalit Helsinki Committee 0195-17-COM2, with exemption from informed consent as the study, is observational and used de-identified data.

### Pedigree and kinship matrix construction

We obtained pedigree information through demographics of past and present patients as well as their parents, and then excluded cases where parental relationships and sex contradicted (e.g., a female father). We converted the entire pedigree to a directed graph using NetworkX^31^, where nodes and edges corresponded to individuals and to parenthood respectively, and removed all edges of directed cycles, as these are not feasible^32^.

Heritability estimates require a kinship matrix, also known as an Additive Relationship Matrix (ARM)^33^, measuring the proportion of identical alleles between pairs of individuals. We approximated the ARM solely from pedigree information, under the assumption that alleles distribute uniformly, meaning each gene has an identical probability to be passed on^34^. For every pair of individuals and a unique shortest path between them through a shared ancestor, we increased their similarity by ${2}^{-l}$ where $l$ is the number of edges in the path (Supplementary Fig. 1a–c).

We decided against removing first-degree relatives in heritability estimates. Although some studies suggest it reduces estimation bias, we found it less relevant to our case^35^.

### Identification of T2D patients

In Israel, T2D is diagnosed based on plasma glucose criteria, in accordance with The American Diabetes Association standard of care^36^. Meeting any of the following criteria is sufficient for T2D diagnosis: (1) random plasma glucose $\ge 200$ mg/dL; (2) HbA1c% $\ge 6.5 \%$; (3) two separate test samples of fasting plasma glucose $\ge 126$ mg/dL following no caloric intake for at least 8 h; (4) plasma glucose $\ge 200$ mg/dL 2 h after oral glucose-tolerance test (OGTT).

Note that although fasting glucose could be used in the diagnosis of T2D, data is inaccurate as some non-fasting patients take the test as well. Also, OGTT tests are not performed regularly in clinics, making us disregard the corresponding criterion.

Due to the nature of the Israeli healthcare system, it is a possibility that an individual was diagnosed with diabetes based on tests unavailable in our database (e.g., in hospital). Therefore, in addition to identifying T2D patients through test results, we made use of diagnoses data. Including all patients diagnosed with T2D according to the appropriate International Classification of Diseases, Ninth Revision (ICD-9) codes^37^ (Supplementary Table 1 and Supplementary Fig. 2).

### Cohort definition

Our cohort constitutes T2D patients treated for diabetes with metformin only after a diabetes diagnosis. We identified those from drug prescriptions with the fifth level Anatomical Therapeutic Chemical (ATC)^38^ code of “A10BA02”. We defined the first metformin prescription date for every patient as index date, yielding a single unique date per individual by which all other dates were measured.

We identified faulty metformin prescriptions consisting of more than three pills per day, and removed information from these prescriptions. We removed from our cohort individuals where the first metformin prescription was faulty.

To establish glycaemic response to metformin we used HbA1c% blood concentration before and after metformin treatment initiation (Supplementary Fig. 1d). We defined baseline (pretreatment) HbA1c% as the latest test occurring 90 days prior to 14 days post index date. This interval was chosen in order to ensure a balance between measurements being within a red blood cell life cycle and metformin’s onset of action, which is within 2 weeks^39^. To ensure stability of results, we estimate heritability on several baseline time intervals for the entire cohort (Supplementary Table 2). We define the on-treatment HbA1c% as the closest test to the index date that is at least 90 days from both index date and baseline HbA1c% date, indicating hemoglobin turning rate. We discarded on-treatment HbA1c% tests later than 180 days from index date, as those are confounded by unmeasured variables. We defined the study participation period as the time from index date or baseline measurement date, whichever preceded, until the on-treatment measurement date.

We ensured measuring the effect of metformin and eliminated cases of initial non-adherence by further screening patients who were treated throughout the entire study participation period^40^. We removed all patients who stopped metformin treatment before on-treatment HbA1c% test or who started taking metformin before being diagnosed with T2D, the majority of which were prescribed metformin while already diagnosed as pre-diabetic. We also exclude all patients who are prescribed any other anti-diabetic medication (ATC level 2 code of ‘A10’) apart from metformin to ensure the effect on HbA1c% levels can be attributed solely to metformin.

We further removed all patients who were diagnosed with type 1 diabetes according to ICD-9 codes (Supplementary Table 3). In addition, we excluded individuals with abnormal estimated Glomerular Filtration Rate (eGFR) who should not be treated according to medical guidelines^41^. GFR is estimated using creatinine blood tests and reflects renal clearance and total clearance, which after oral administration of metformin decrease approximately in proportion to it^42^ (Table 1).

**Table 1:** Inclusion exclusion criteria.

Inclusion	Exclusion
Type 2 diabetic treated with metformin only after diagnosis.	Abnormal estimated glomerular filtration rate (eGFR<30 mL/min/1.73 m^2^)
Baseline HbA1c% test exists 90 days prior to 30 days after first metformin prescription	Treated for diabetes with non-metformin drugs (ATC2 is “A10”)
On-treatment HbA1c% test exists 90–180 days after first metformin prescription	Baseline HbA1c% and on-treatment HbA1c% tests <90 days apart

### Glycaemic response outcomes

We defined three phenotypes commonly used in metformin pharmacogenetics studies for measuring the response to metformin; absolute, proportional, and adjusted reduction in HbA1c%^16^. These were induced from the difference between the baseline and the on-treatment HbA1c% tests. The absolute reduction was defined as the absolute difference between on-treatment and baseline HbA1c%, proportional reduction was defined as the absolute reduction divided by the baseline HbA1c%. We trained a linear model to predict absolute reduction from pretreatment HbA1c% measurement, the number of days between pretreatment and on-treatment HbA1c% measurement dates and average metformin dose during the study (see further explanation below). The adjusted reduction was defined as the residuals from the linear model’s predicted phenotype to the true absolute reduction values. Since Linear Mixed Models assume normal distribution we performed the Kolmogorov–Smirnov goodness of fit test for all three phenotypes^43,44^.

### Height outcome

Being that the heritability estimate of height is well established and agreed upon in the literature; we used it as a positive control to validate our methods and data. We gathered height measurements recorded at adulthood (age $\ge$18 years). For patients who had multiple measurements, we considered the latest measurement only. We removed outlier measurements where *Z* score>4.

### Heritability estimation

We computed heritability with the Sci-LMM Python package, which constructs and works with large-scale relationships matrices and fits them to the corresponding LMM within several hours. Our Identity By Descent (IBD) matrix (an identity-by-descent relationships-based matrix) was the ARM computed from the entire pedigree^28^. We used Haseman-Elston regression to compute the heritability measure ${h}^{2}$, and we estimate the standard error via the average information restricted maximum likelihood (AI-REML) procedure^45,46^.

We constructed the following features used either as covariates for our regression model or as means of subsampling the cohort:

1. Demographics:
  - Year of birth
  - Age at index date
  - Gender
  - BMI: note that since is considered heritable we did not use it as a covariate in our regression.
2. Measurements’ metadata:
  - Baseline to index gap: number of days between baseline date to index date
  - Index to on-treatment gap: number of days between index date and on-treatment date
  - Baseline to on-treatment gap: number of months between on-treatment date and baseline date. Note that due to co-linearity with the two previous covariates, this covariate was not in use.
  - Number of HbA1c% tests: the absolute number of HbA1c% tests performed up until the on-treatment date
3. Lab test measurements:
  - Estimated glomerular filtration rate (eGFR): We used MDRD GFR Equation:^47^ ${{{{{{\mathrm{eGFR}}}}}}}=186\times {{{{{{{\mathrm{creatinine}}}}}}}}^{-1.154}\times {{{{{{\mathrm{age}}}}}}}^{-0.203}$ where value is multiplied by 0.742 for females.
  - Baseline HbA1c%
4. Treatment metadata:
  - Average dosage: weighted average of metformin doses $\varSigma {w}_{i}\times {p}_{i}/\varSigma {p}_{i}$ where ${w}_{i}$ is the number of pills per day prescribed in prescription $i$, and ${p}_{i}$ is the number of pills in prescription $i$. Only issued prescriptions were accounted for.
  - Adherence: since adherence is not reported, we capture it through four features representing the average number of days on metformin in four equal consecutive time intervals between index date and on-treatment date. We assumed that all dispensed prescriptions were also consumed by patients.

In order to identify environmental variance, we computed the explained variance from only the covariates. We trained a linear regressor from covariates predicting absolute reduction. We had then computed the Pearson’s correlation of predicted and true reduction as well as the ${R}^{2}$ score.

### Predicting outcome

We assessed the predictive potential family history could give to treatments, we predicted responses to metformin from both covariates and family information. We constructed family history features for each individual by computing mean absolute reduction from relatives. We computed four features considering either all relatives or only relatives from the same gender as the individuals, and taking only first-degree relatives or all available relatives. We predicted on-treatment HbA1c% for the entire cohort with the above-mentioned covariates, excluding adherence, as it is only available while on-treatment. Predictions were performed using XGBoost regression with 100-fold cross-validation and n_estimators=20^48^. We computed the mean squared error (MSE) on predicted outcome for the entire cohort as well as for only individuals who have any relatives within the cohort. We also predicted for these individuals the outcome using both covariates and family history features.

### Statistics and reproducibility

All statistics were performed using Python 3.7 software. Statistical significance was determined by mentioned unpaired tests using Scipy 1.3.1. Experiments are reproducible with existing EHR records; however, this work was performed on data from Clalit Health Services which is not publicly available. Sample sizes are defined within the work.

### Reporting summary

Further information on research design is available in the Nature Research Reporting Summary linked to this article.

## Results

### Cohort description

**Fig. 1:**
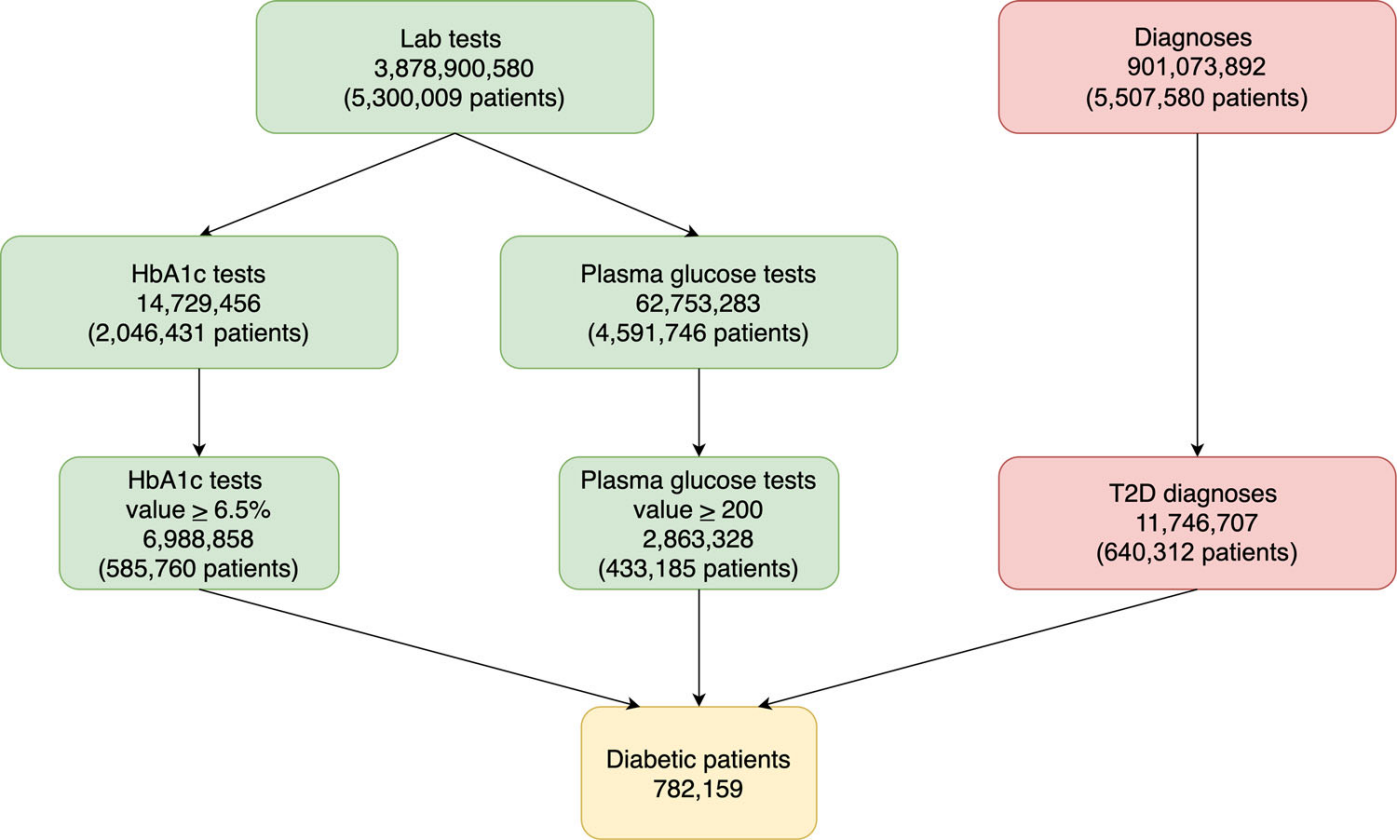
T2D patients’ selection. Patients were determined by fulfilling at least one of three criteria: (1) HbA1c% lab test value>6.5%; (2) plasma glucose lab test value>200 mg/dL; (3) T2D diagnosis. Green applies to lab test results, red to diagnoses, and yellow to patients. In total, 782,159 T2D patients were identified.

To estimate the heritability measure of response to metformin we extracted 782,159 T2D patients from the Clalit Healthcare EHR database (Fig. 1). Of these, we included only patients who had at least two HbA1c% test measurements, one before metformin treatment (baseline) and one after it (on-treatment). We excluded subjects treated with non-metformin diabetic drugs prior to the on-treatment date, and those treated with metformin prior to T2D diagnosis, maintaining a total of 80,788 patients.

In total, our cohort was balanced between genders, with 49% males. When comparing feature distribution between genders we found they all differ significantly, with the exception of eGFR and adherence on the second time interval (e.g., average ages were 59.90 for males and 61.65 for females) (Table 2). We see therefore that although the two populations differ on every parameter, they receive similar treatments. Since they are all T2D patients, our cohort diverts from the general population by risk factors characteristics. In addition to being older and having, an obese BMI on average, patients had a baseline HbA1c% of 7.73% for males and 7.34% for females, ~1% over diabetic threshold HbA1c% value (6.5%). We identified that 33.7% T2D patients had at least one first-degree relative diagnosed with T2D. These patients had 1.88 first-degree relatives on average also diagnosed with T2D.

**Table 2:** Baseline characteristics of the study cohort.

	Gender	Mean	STD	Median	FDR-corrected *P* value*	Availability, %
Demographics						
Age, years	F	61.65	12.38	62	2E-89	100
	M	59.9	12.38	60		100
BMI, Kg/m^2^	F	32.41	7.02	31.54	<1E-350	82.95
	M	30.36	6.04	29.65		83.17
Gender (is male)	Joint	0.49	0.5	0		100
Year of birth	F	1948.78	13.15	1949	1E-106	100
	M	1950.82	13.18	1951		100
Lab tests measurements						
Baseline HbA1c%	F	7.34	1.34	7	4E-290	100
	M	7.73	1.64	7.2		100
eGFR**	Joint	93.54	24.5	90.95		87.47
Measurements’ metadata						
Number of HbA1c% measurements	F	6.01	4.54	5	2E-27	100
	M	5.67	4.39	4		100
Base to index gap, days	F	18.93	21.55	11	1E-42	100
	M	16.89	20.69	9		100
Base to on-treatment gap, days	F	150.09	34.69	147	1E-29	100
	M	147.33	34.25	145		100
Index to on-treatment gap, days	F	131.16	26.53	129	0.0002	100
	M	130.45	26.56	128		100
Treatment metadata						
Adherence 1, %	F	92.84	12.06	100	0.002	100
	M	93.11	11.92	100		100
Adherence 2, %	Joint	58.24	38.84	70.73	0.02	100
Adherence 3, %	F	55.69	40.4	69.05		100
	M	55.01	40.29	66.67		100
Adherence 4, %	F	53.9	40.56	65.52	0.0002	100
	M	52.8	40.42	62.5		100
Average dose, Mg	F	1.32	0.52	1	2E-107	100
	M	1.4	0.56	1		100

*P value for in ttest comparing male and female distributions. Appears only when the difference in distributions is significant.

**eGFR is in units defined in the formula above.

Availability of “Average dose while taking” and “Adherence” was calculated through the percent of available prescriptions, which were used to generate these features.

### Height heritability estimation

We first set out to validate our framework through estimation of height heritability, as it is a well-documented heritable measure^49^. Such validation indicates whether the constructed pedigree could be used for heritability estimation, and whether Clalit’s EHR population is representative of the general population. Our estimation took into account two covariates, sex and year of birth, both of which are highly correlated with height regardless of heritable effects^50^.

We extracted height measurements of 11,466,686 adults from 5,275162 families with the largest family consisting of 4,157,673 adults. A Kolmogorov–Smirnov test indicated that height followed a normal distribution (Supplementary Table 4) with a mean of 1.68 m and a standard deviation of 0.10 m (Table 3). We estimated the heritability measure of the height to be ${h}^{2}=80.0 \%$ (95% CI 79.2–80.8%), a value that is consistent with the literature^49^, thus validating both our approach and our dataset.

**Table 3:** Cohort Phenotypes Statistics.

	Gender	Mean	Standard deviation	Median	FDR-corrected *P* value
Height, m	F	1.62	0.07	1.62	<E-350
	M	1.75	0.07	1.75	
	Joint	1.68	0.10	1.68	
HbA1c% absolute reduction	F	0.67	1.20	0.40	7E-308
	M	1.04	1.57	0.60	
	Joint	0.85	1.40	0.50	
HbA1c% adjusted reduction	F	−0.16	0.92	−0.21	5E-121
	M	0.01	1.12	−0.09	
	Joint	−0.08	1.03	−0.15	
HbA1c% proportional reduction	F	0.08	0.12	0.06	1E-303
	M	0.11	0.15	0.08	
	Joint	0.09	0.14	0.07	

### Metformin response characteristics

We computed three outcomes of response to newly metformin-treated T2D patients from HbA1c% reduction: absolute, adjusted, and proportional. We find that the mean absolute HbA1c% reduction is 0.85%, which concurs with known reductions after first-time metformin treatment^51^. It is important to note that response depends on treatment policy, as individuals with higher baseline HbA1c% receive higher doses of metformin which in turn result in larger HbA1c% reductions.

When observing the different phenotypes, we find that they all differ significantly between males and females (Table 3). We have also found all phenotypes to have statistically significant different variances (Supplementary Table 5). This led us to compute heritability estimates for each sex independently. Furthermore, we decided to compute estimates for additional subgroups within the population to better understand their independent heritable effect on metformin response.

### Heritability of response to metformin

We computed the heritability of HbA1c% reduction phenotypes on the cohorts of 39,335 male patients and 41,453 female patients. For our calculations, we used covariates of personal measures as well as treatment strategy measures (see “Methods”). We found that the heritability measure of absolute HbA1c% reduction is ${h}^{2}=12.6 \%$ (95% CI, 6.1–19.1%) for the entire cohort, ${h}^{2}=21.0 \%$ (95% CI, 7.8–34.3%) for males and ${h}^{2}=22.9 \%$ (95% CI, 10.0–35.7%) for females (Table 4).

**Table 4:** Heritability estimates of metformin responses.

Cut	HbA1c absolute reduction		HbA1c-adjusted reduction		HbA1c proportional reduction		Number of patients
	*h*^2^	CI	*h*^2^	CI	*h*^2^	CI	
All cohort	0.126	[0.061, 0.191]	0.126	[0.061, 0.191]	0.138	[0.073, 0.203]	80,788
Male	0.21	[0.078, 0.343]	0.21	[0.078, 0.343]	0.232	[0.099, 0.364]	39,335
Female	0.229	[0.1, 0.357]	0.229	[0.1, 0.357]	0.223	[0.094, 0.351]	41,453

*h*^2^ estimates and their confidence intervals of our cohort including different subgroups.

We examined whether our heritability estimates remain similar between different metformin response phenotypes. We found that the heritability estimates for the adjusted HbA1c% reductions are identical for all groups. This result is expected, as the adjusted reduction values are the residuals from a model that is based on covariates. We find that the estimates for proportional HbA1c% reduction are also relatively similar and are ${h}^{2}=13.8 \%$ (95% CI, 7.3–20.3%) for the entire cohort, ${h}^{2}=23.2 \%$ (95% CI, 9.9–36.4%) for males and ${h}^{2}=22.3 \%$ (95% CI, 9.4–35.1%) for females. We note that across the three phenotypes the 95% intervals of estimates overlap, demonstrating that the estimates are relatively similar.

We estimated the explained variance of the covariates alone without family information to identify the explained variance of environmental factors. We computed the explained variance of absolute reduction in HbA1c% as 66.6%.

We estimated the heritability of responses to additional subgroups of the cohort, in order to search for affecting factors. We split our cohort by age (binning them by decades), and by absolute HbA1c% response values as well as by ethnicity, and found no meaningful results most likely due to the small sizes of those subgroups (Supplementary Table 6).

### Predicted response to metformin

We predicted the on-treatment HbA1c% with on the entire cohort of 80,788 individuals reaching an MSE of $1.2$. We found a total of 8,075 individuals with relatives within the cohort. The MSE of the prediction on said individuals was $1.6$. When predicting from both covariates and family history features, we computed an MSE of $1.4$.

## Discussion

In this work, we estimated the heritability of response to metformin treatment in patients with T2D. Our cohort consists of 782,159 patients with T2D, 80,788 of whom begin metformin treatment while already recorded in the EHRs. In combination with pedigree information from national registries, we constructed a kinship matrix yielding genetic similarities between all patients. From it we estimated the heritable component of absolute reduction in HbA1c% following metformin in newly treated patients to be $${h}^{2}=12.6 \%$$ (95% CI, 6.1–19.1%) for both genders, $${h}^{2}=21.0 \%$$ (95% CI, 7.8–34.3%) for males and $${h}^{2}=22.9 \%$$ (95% CI, 10.0–35.7%) for females. This value remained unchanged when adjusting the response for pretreatment personal covariates and for proportional HbA1c% reduction compared to baseline HbA1c%. The similarity of results is most likely due to the correlation between proportional and absolute reductions $$r=0.97$$ (*P* value$$ < 6E-310$$) (Supplementary Fig. 3).

In metformin-based studies, EHR data are usually leveraged to explore its repurposing to other diseases, or to estimate its individualized treatment effect^28,52^. Common approaches for estimating drug response heritability compute genetic similarities through genetic tests^16,53–55^. Collecting genetic information leads to small cohorts and requires international collaborations. Our study obtained inherited similarities between individuals from national registries, these hold promise due to their tremendous size, and have been previously employed to estimate heritability measures of longevity, autism, and others^28,52^.

To the best of our knowledge, our study is the first to assess the heritability of variation to drug response by fitting an LMM solely from EHR data combined with a pedigree-based IBD matrix, as the model’s kinship matrix. We validated the proposed method by estimating the heritability of adult height, finding the measure to be ${h}^{2}=80.0 \%$ (95% CI 79.2–80.8%). This result agrees well with the widely accepted heritability of height of 80%^56^, thus strengthening our belief that our use of pedigrees in heritability estimates is robust to non-biological noise as well as to possible inaccuracies in the EHR pedigree (either wrong or missing information). This conclusion, that heritability estimation from EHR is a valid methodology is consistent with the previous studies^57^. Furthermore, it does not require patient recruitment as well as costly genetic tests. We estimated the heritability of metformin responses to be in the range of 10–20%, suggesting that while genetics likely contribute to variation in metformin glycaemic response for T2D patients, most of the variation is likely due to other environmental factors.

Estimated metformin responses heritability measures are within the parameters of previous genetic-based estimations, however with smaller confidence intervals^16^. The increased statistical power is a direct result of our relatively large cohort size compared with previous works that commonly consist of up to several thousand patients^16,54,55^.

Distinguishing between genetic and environmental effects is often difficult and not impossible. For example, when prescribing metformin, physicians also commonly advise lifestyle changes. These changes, if followed, can have a positive effect on the reduction of HbA1c levels, which in our study are attributed solely to metformin. We note that most individuals in our cohort were prescribed metformin at an older age (average age at index date is 60), and since our analyses show a gradual increase in HbA1c we presume that most individuals were advised to make lifestyle changes prior to their first metformin prescription. Nevertheless, in our case, pedigree data encompasses more than just genetic information, as it provides some underlying information of environmental factors, especially in the case of first-degree relatives. Although this makes results more difficult to decipher, the accurate results of our positive and negative controls provide confidence in our method. We, therefore, believe that the included covariates capture the majority of the environmental variance and hence, prevent their effect on our ${h}^{2}$ estimates. In spite of our efforts, we believe that it is still possible that some passed environmental information remains in our heritability estimates.

To ensure that we only account for the effect of metformin, we excluded from our cohort patients treated with other anti-diabetic drugs. However, we did not include covariates of other drugs that may interact with metformin and alter its effect. In addition, we performed our analysis on dispensed metformin prescriptions with the underlying assumption that to an extent, it is an indicator for adherence. Although eliminating cases of initial non-adherence to the best of our ability, we assume some level of non-adherence to affect our results and cause biases in our estimations. We note that the vast majority of individuals in our study were prescribed at least three different prescriptions of 30 pills each in the course of 90 days, suggesting they are likely to have consumed the metformin. This is one of the limitations of working with EHR data compared to the much more controlled setting of randomized clinical trials. On the other hand, our analysis depicts real-world scenarios and may thus provide more relevant estimates for the true underlying effect.

Our results show differences in the heritability of metformin responses when estimated on the entire population or separated by gender, with higher heritability measures for the split model. The joint model, by design, assumes a different mean between genders (encompassed in the gender covariate). On the other hand, the separated models make no assumption on the relationships between the responses of females and males. We show that most covariates differ between the genders, moreover, we also show that all outcomes have statistically significant variances, consistent with results from other countries^58^. These results show that the shared model is prone to higher noise caused by the differences between the two genders. We believe that the inability of the joint estimate to model these differences cause the estimate to be significantly lower. Our analyses also included various stratifications such as age, and baseline HbA1c measurements, for which we found no significant difference in the ${h}^{2}$ estimate or did not have a sufficient amount of data. However, we acknowledge that while we assume in this study that T2D is a homogenous disease, individuals in our cohort likely suffer from a variety of diseases all grouped under the term T2D and resulting in different metabolic defects. In spite variation, our data are limited to the ICD-9 diagnosis codes and does not contain this information, but it is likely that the heritability of glycemic response for metformin varies across individuals suffering from such diseases.

Creating a personalized tailored treatment to T2D patients holds great potential, such treatments could be based on both environmental and genetic factors, and help to faster divert non-responding patients to second-line treatments with less deterioration. Several predictors for second-line treatments already exist, but most do not yield personalized recommendations^59–61^. We show that with the use of a tree model predictions can be performed with an MSE of 1.2 in predicting on-treatment HbA1c%, and that for individuals with family history information MSE improves from 1.6 to 1.4 upon addition of family information features. Our work suggests that future works aiming to estimate metformin effects should probably include family medical history, yet be based mostly on environmental factors. In general, knowledge of drug response heritability like the one presented in this study is a first step in allocating efforts of personalizing treatments, giving an upper bound to the possible effect of family history information.

Overall, our results indicate that while genetics likely contribute to variation in metformin glycaemic response for T2D patients, environmental factors likely have a larger effect. Such findings are in line with prior evaluations of associations between single-nucleotide polymorphisms and the reduction in HbA1c% after introduction to metformin^10^. Our results emphasize the need for personalized treatment regimens of metformin. More generally, our work shows the utility of carrying out pharmacogenetic studies using EHRs, which may yield valuable insights without the burden and cost of genetic tests.

## Supplementary information

Supplementary Information

Reporting Summary

## Data Availability

The data that support the findings of this study originate from Clalit Healthcare Services. All data analyses were conducted on a secured, de-identified dedicated server within the Clalit Healthcare environment. Requests for access to all of parts of the Clalit datasets should be addressed to Clalit Healthcare Services, via the Clalit Research Institute.

## Code availability

The code that supports the findings of this study is tailored to the EHR data in the Clalit Health Services database and is thus not provided since it is of no use as a standalone without access to the data per se. Requests for software modules as a basis for adaptation to other EHR environments or usage of the software on Clalit data following Clalit approval should be addressed to the authors after data access is approved by the Clalit Data Access committee. All analyses used the following open-source publicly available Python (3.7) code packages: Scipy (version 1.3.1), Numpy (version 1.17.0), Pandas (version 0.24.2), Dask (version 2.9.1), Sci-LMM (version 0.1.1).
