# Supplementary material for: Estimating heritability of glycaemic response to metformin using nationwide electronic health records and population-sized pedigree
Source: Commun Med (Lond). 2021 Dec 1;1:55. doi: 10.1038/s43856-021-00058-4 (PMC9053254; doi:10.1038/s43856-021-00058-4)
Supplement: Supplementary file 1 — Supplementary Information [file 43856_2021_58_MOESM1_ESM.pdf]

Supplementary:

Supplementary Figure 1

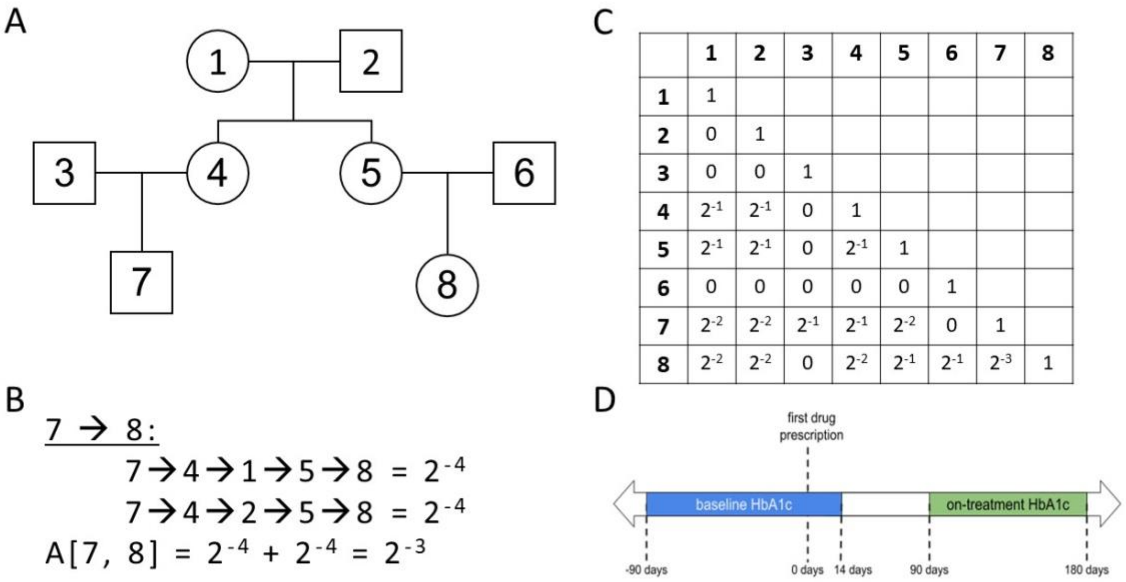

Supplementary Figure 1: Methods overview. *(a) Illustration of an example pedigree. Sex is distinguished by node shape (circle for women and squares for men). (b) Computation of genetic correlation between individuals 7 and 8 from the pedigree. A valid path has to go through a single shared ancestor and not repeat any parent-child edges. There are exactly two unique paths between 7 and 8, both with a total of 4 edges, adding* 2^{-4} *each to the similarity between 7 and 8. (c) The complete additive relationship matrix corresponding to the pedigree. The matrix is symmetric, hence we only display below diagonal entries. (d) Baseline HbA1c% is defined as the latest HbA1c% test that occurred no earlier than 90 days prior, and no later than 14 days from to index-date. On-treatment HbA1c% is defined as the closest test to the index-date that is at least 90 days from index-date and at least 90 days from the baseline HbA1c% date, but no later than 180 days from index-date*.

	1	2	3	4	5	6	7	8
1	1							
2	0	1						
3	0	0	1					
4	2^{-1}	2^{-1}	0	1				
5	2^{-1}	2^{-1}	0	2^{-1}	1			
6	0	0	0	0	0	1		
7	2^{-2}	2^{-2}	2^{-1}	2^{-1}	2^{-2}	0	1	
8	2^{-2}	2^{-2}	0	2^{-2}	2^{-1}	2^{-1}	2^{-3}	1

Supplementary Figure 2

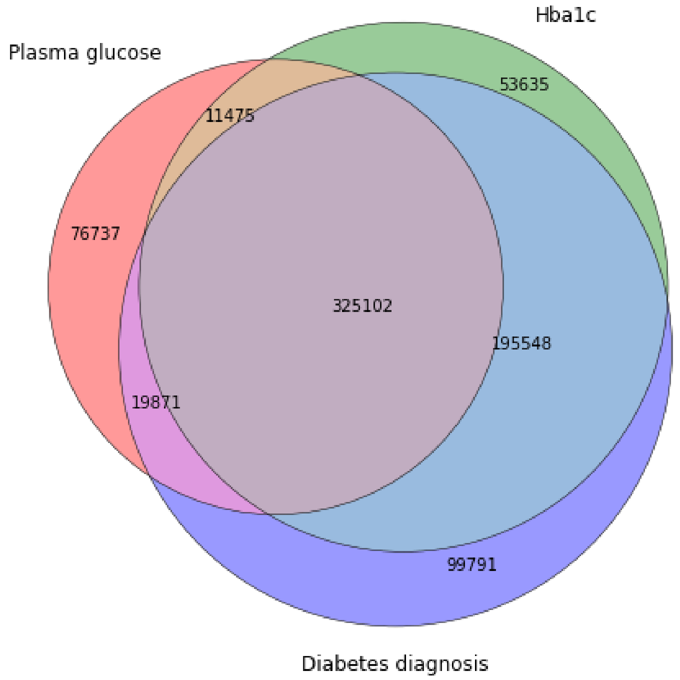

Supplementary Figure 2: Origin of T2D. *Venn diagram of the source by which we defined individuals as having T2D. Three origins exist: plasma glucose, HbA1c measurements or diabetes diagnosis from ICD-9 codes. In this figure, we defined individuals to have T2D from an origin if they had any such origin, regardless of the date.*

Supplementary Figure 3

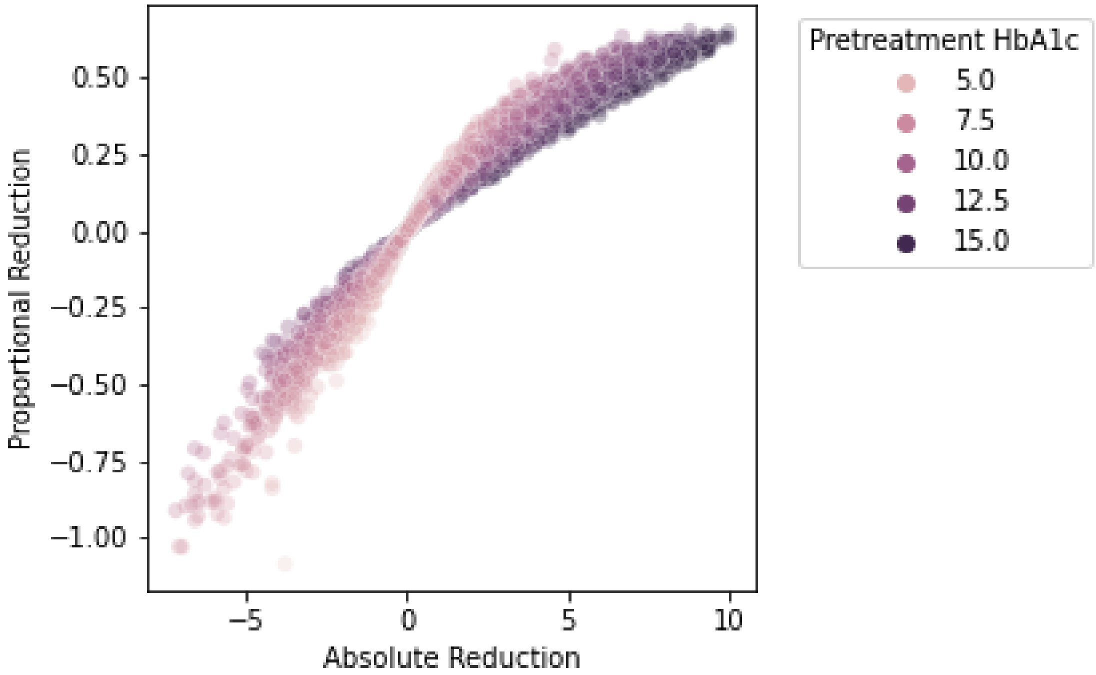

Correlation of HbA1c% absolute and proportional reduction. Scatter of reductions, each point represents a single individual from the cohort. Colors of points are the pretreatment HbA1c% values. Overall, the Pearson's correlation is $r = 0.97$ $(p - value < 6E - 310)$.

Supplementary Table 1

ICD-9	Description
250	DIABETES MELLITUS
2500	DIABETES MELLITUS WITHOUT MENTION OF COMPLICATION
2501	DIABETES WITH KETOACIDOSIS
2502	DIABETES WITH HYPEROSMOLAR COMA
2503	DIABETES WITH OTHER COMA
2504	DIABETES WITH RENAL MANIFESTATIONS
2505	DIABETES WITH OPHTHALMIC MANIFESTATIONS
2506	DIABETES WITH NEUROLOGICAL MANIFESTATIONS
2507	DIABETES WITH PERIPHERAL CIRCULATORY DISORDERS
2508	DIABETES WITH OTHER SPECIFIED MANIFESTATIONS
25080	ADULT-ONSET DIABETES MELLITUS WITH SPECIFIED MANIFEST.
25092	DIABETES + UNSP. COMPLICATION, TYPE II, UNCONTROLLED
25090	ADULT-ONSET TYPE DIABETES MELLITUS WITH UNSPECIFIED COMPLICATION
2509	DIABETES WITH UNSPECIFIED COMPLICATION
25082	ADULT-ONSET DIABETES, UNCONTROLLED, + SPEC. MANIFESTATIONS
25072	DIABETES+PERIPHERAL CIRCULATORY DISORDERS, TYPE II, UNCONTROLLED
25070	ADULT-ONSET DIABETES MELLITUS WITH PERIPH.CIRCUL.DISORDERS
25062	DIABETES WITH NEUROLOGICAL MANIFESTATIONS, TYPE II, UNCONTROLLED
25060	ADULT-ONSET DIABETES MELLITUS WITH NEUROLOGICAL MANIFEST.
25052	ADULT-ONSET DIABETES, UNCONTROLLED,+ OPHTHALMIC MANIFESTATIONS
25050	ADULT-ONSET DIABETES MELLITUS WITH OPHTHALMIC MANIFEST.
25042	DIABETES WITH RENAL MANIFESTATIONS, TYPE II, UNCONTROLLED
25040	ADULT-ONSET TYPE DIABETES MELLITUS WITH RENAL MANIFESTATIONS
25032	ADULT-ONSET DIABETES, UNCONTROLLED, WITH OTHER COMA
25030	ADULT-ONSET TYPE DIABETES MELLITUS WITH OTHER COMA
25022	ADULT-ONSET DIABETES, UNCONTROLLED, WITH HYPEROSMOLARITY
25020	ADULT-ONSET TYPE DIABETES MELLITUS WITH HYPEROSMOLAR COMA
25012	DIABETES WITH KETOACIDOSIS, TYPE II, UNCONTROLLED
25010	ADULT-ONSET TYPE DIABETES MELLITUS WITH KETOACIDOSIS
25002	DIABETES MELLITUS WITHOUT COMPLICATION, TYPE II, UNCONTROLLED

25000	ADULT-ONSET TYPE DIABETES MELLITUS WITHOUT COMPLICATION

ICD-9 codes of T2D. Above are ICD-9 codes and descriptions included as T2D diagnoses. These are all codes starting with 250 and including the term “diabetes”. We removed diagnoses of type 1 by excluding codes where either “type I” or “juvenile” appeared in description.

Supplementary Table 2

Maximum pretreatment index-date days	Number of patients	Absolute HbA1c% reduction		Adjusted HbA1c% reduction		Proportional HbA1c% reduction	
		h2	CI	h2	CI	h2	CI
30	64248	0.151	[0.07, 0.233]	0.151	[0.07, 0.233]	0.158	[0.076, 0.239]
60	75234	0.148	[0.078, 0.218]	0.148	[0.078, 0.218]	0.161	[0.091, 0.231]
90	80868	0.120	[0.055, 0.184]	0.120	[0.055, 0.184]	0.133	[0.068, 0.198]
365	89869	0.101	[0.042, 0.159]	0.101	[0.042, 0.159]	0.118	[0.059, 0.177]

Heritability measures for changing time intervals. Shown are the heritability estimates for the entire cohort with a changing difference between pretreatment and treatment days. We see that results are aligned with main ones. For a yearlong, we see lower heritability, which is likely the result of more noise in data.

Supplementary Table 3

ICD-9	Description
25093	DIABETES + UNSP. COMPLICATION, TYPE I, UNCONTROLLED
25091	JUVENILE TYPE DIABETES MELLITUS WITH UNSPECIFIED COMPLICATION
25083	JUVENILE DIABETES, UNCONTROLLED, + SPEC. MANIFESTATIONS
25081	JUVENILE DIABETES MELLITUS WITH SPECIFIED MANIFEST.
25073	DIABETES+PERIPHERAL CIRCULATORY DISORDERS, TYPE I, UNCONTROLLED
25071	JUVENILE DIABETES MELLITUS WITH PERIPH.CIRCULAT.DISORDERS
25063	DIABETES WITH NEUROLOGICAL MANIFESTATIONS, TYPE I, UNCONTROLLED
25061	JUVENILE DIABETES MELLITUS WITH NEUROLOGICAL MANIFEST.
25053	JUVENILE DIABETES, UNCONTROLLED, + OPHTHALMIC MANIFESTATIONS
25051	JUVENILE TYPE DIABETES MELLITUS WITH OPHTHALMIC MANIFESTATIONS
25043	DIABETES WITH RENAL MANIFESTATIONS, TYPE I, UNCONTROLLED
25041	JUVENILE TYPE DIABETES MELLITUS WITH RENAL MANIFESTATIONS
25033	JUVENILE DIABETES, UNCONTROLLED, WITH OTHER COMA
25031	JUVENILE TYPE DIABETES MELLITUS WITH OTHER COMA
25023	JUVENILE DIABETES, UNCONTROLLED, WITH HYPEROSMOLARITY
25021	JUVENILE TYPE DIABETES MELLITUS WITH HYPEROSMOLAR COMA
25013	DIABETES WITH KETOACIDOSIS, TYPE I, UNCONTROLLED
25011	JUVENILE TYPE DIABETES MELLITUS WITH KETOACIDOSIS
25003	DIABETES MELLITUS WITHOUT COMPLICATION, TYPE I, UNCONTROLLED
25001	JUVENILE TYPE DIABETES MELLITUS WITHOUT MENTION OF COMPLICATION

ICD-9 codes of T1D. Above are ICD-9 codes and descriptions included as T1D diagnoses. These are all codes starting with 250 and including the term “diabetes” as well as either the term “type I” or “juvenile” in their description.

Supplementary Table 4

	D	p-value
**Height**	0.92	<1E-230
**HbA1c absolute reduction**	0.34	<1E-230
**HbA1c proportional reduction**	0.43	<1E-230
**HbA1c adjusted reduction**	0.09	<1E-230

Kolmogorov-Smirnov results on target values. Linear Mixed Models assume normal distribution of target values. Shown here are the results of goodness of fit Kolmogorov-Smirnov tests on outcomes. From it, we conclude that all targets are normally distributed with high probability. For the height outcome N=11,466,686, for other outcomes N=80,868.

Supplementary Table 5

	F	p-value
HbA1c absolute reduction	1.73	<E-266
HbA1c proportional reduction	1.56	<E-266
HbA1c adjusted reduction	1.48	<E-266

Variance comparison between genders. F-test results comparing metformin response outcomes between males and females. Number of individuals: females=41,453, males=39,335.

Supplementary Table 6

		Number of patients	Absolute reduction		Adjusted reduction		Proportional reduction	
Subset type	Groups		h^2	CI	h^2	CI	h^2	CI
Age	10-19	4						
	20-29	130						
	30-39	611	-1.639	[-3.206, -0.072]	-1.639	[-3.206, -0.072]	-1.485	[-3.003, 0.033]
	40-49	3677	-0.156	[-0.602, 0.289]	-0.156	[-0.602, 0.289]	0.009	[-0.439, 0.457]
	50-59	11801	0.359	[0.15, 0.568]	0.359	[0.15, 0.568]	0.366	[0.157, 0.575]
	60-69	23115	-0.070	[-0.292, 0.152]	-0.070	[-0.292, 0.152]	-0.077	[-0.299, 0.145]
	70-79	23061	0.240	[-0.357, 0.837]	0.240	[-0.357, 0.837]	0.074	[-0.515, 0.664]
	80-89	14161						
	90-99	3940						
	100-109	284						
	110-119	4						
Absolute HbA1c% reduction	(-7.3, 0.1]	20637	0.317	[0.078, 0.556]	0.317	[0.078, 0.556]	0.291	[0.053, 0.53]
	(0.1, 0.5]	23366	-0.097	[-0.336, 0.142]	-0.097	[-0.336, 0.142]	-0.134	[-0.372, 0.105]
	(0.5, 1.1]	17066	-0.037	[-0.35, 0.275]	-0.037	[-0.35, 0.275]	0.019	[-0.293, 0.332]
	(1.1, 10.0]	19719	0.243	[0.032, 0.455]	0.243	[0.032, 0.455]	0.296	[0.083, 0.509]
Ethnic Groups	Europe	22837	0.021	[-0.174, 0.216]	0.021	[-0.174, 0.216]	0.010	[-0.185, 0.205]
	Africa	15126	0.155	[0.005, 0.305]	0.155	[0.005, 0.305]	0.165	[0.015, 0.315]
	Middle East	13223	0.166	[0.007, 0.326]	0.166	[0.007, 0.326]	0.178	[0.019, 0.338]
	America	1587	-1.147	[-2.073, -0.221]	-1.147	[-2.073, -0.221]	-1.013	[-1.914, -0.112]

Heritability measures of cuts. Heritability measures as calculated on different cuts. Missing values are caused due to small cohort sizes or lack of connectivity between individuals. We note that values outside the 0-1 interval are possible in the estimations but are not feasible in reality, yet they are reported here.
