# Supplementary material for: Estimating heritability of glycaemic response to metformin using nationwide electronic health records and population-sized pedigree
Source: Commun Med (Lond). 2021 Dec 1;1:55. doi: 10.1038/s43856-021-00058-4 (PMC9053254; doi:10.1038/s43856-021-00058-4)
Supplement: Supplementary file 2 — Reporting Summary [file 43856_2021_58_MOESM2_ESM.pdf]

nature research

Corresponding author(s): COMMSMED-21-0133B

Last updated by author(s): Oct 31, 2021

Reporting Summary

Nature Research wishes to improve the reproducibility of the work that we publish. This form provides structure for consistency and transparency in reporting. For further information on Nature Research policies, see our Editorial Policies and the Editorial Policy Checklist.

Statistics

For all statistical analyses, confirm that the following items are present in the figure legend, table legend, main text, or Methods section.

n/a	Confirmed	
☐	☒	The exact sample size (*n*) for each experimental group/condition, given as a discrete number and unit of measurement
☐	☒	A statement on whether measurements were taken from distinct samples or whether the same sample was measured repeatedly
☐	☒	The statistical test(s) used AND whether they are one- or two-sided<br>*Only common tests should be described solely by name; describe more complex techniques in the Methods section.*
☐	☒	A description of all covariates tested
☐	☒	A description of any assumptions or corrections, such as tests of normality and adjustment for multiple comparisons
☐	☒	A full description of the statistical parameters including central tendency (e.g. means) or other basic estimates (e.g. regression coefficient) AND variation (e.g. standard deviation) or associated estimates of uncertainty (e.g. confidence intervals)
☐	☒	For null hypothesis testing, the test statistic (e.g. *F*, *t*, *r*) with confidence intervals, effect sizes, degrees of freedom and *P* value noted<br>*Give P values as exact values whenever suitable.*
☒	☐	For Bayesian analysis, information on the choice of priors and Markov chain Monte Carlo settings
☒	☐	For hierarchical and complex designs, identification of the appropriate level for tests and full reporting of outcomes
☒	☐	Estimates of effect sizes (e.g. Cohen's *d*, Pearson's *r*), indicating how they were calculated

Our web collection on statistics for biologists contains articles on many of the points above.

Software and code

Policy information about availability of computer code

Data collection	No data collection software was performed.
Data analysis	Python 3.7 was used with the following packages:<br>- Scipy (version 1.3.1)<br>- Numpy (version 1.17.0)<br>- Pandas (version 0.24.2)<br>- Dask (version 2.9.1)<br>- SciLMM (version 0.1.1)

For manuscripts utilizing custom algorithms or software that are central to the research but not yet described in published literature, software must be made available to editors and reviewers. We strongly encourage code deposition in a community repository (e.g. GitHub). See the Nature Research guidelines for submitting code & software for further information.

Data

Policy information about availability of data

All manuscripts must include a data availability statement. This statement should provide the following information, where applicable:

- Accession codes, unique identifiers, or web links for publicly available datasets
- A list of figures that have associated raw data
- A description of any restrictions on data availability

The data that support the findings of this study originate from Clalit Health Services. Restrictions apply to the availability of these data and they are therefore not publicly available. Due to restrictions, these data can be accessed only by request to the authors and/or Clalit Health Services.

Field-specific reporting

Please select the one below that is the best fit for your research. If you are not sure, read the appropriate sections before making your selection.

☒ Life sciences ☐ Behavioural & social sciences ☐ Ecological, evolutionary & environmental sciences

For a reference copy of the document with all sections, see nature.com/documents/nr-reporting-summary-flat.pdf

Life sciences study design

All studies must disclose on these points even when the disclosure is negative.

Sample size	Our study was performed on electronic health records of over 5 million people. From them we identified 80,788 of newly treated type 2 diabetes patients who were treated solely with metformin.
Data exclusions	Data exclusion was performed by removing all lab test results with absolute z-score greater than 4. Metformin perscriptions were excluded in cases where number of pills per day excludes 3 (guidline values).
Replication	Replication is not relevant in a retrospective observational study.
Randomization	Randomization is not relevant in a retrospective observational study.
Blinding	Blinding is not relevant in a retrospective observational study.

Reporting for specific materials, systems and methods

We require information from authors about some types of materials, experimental systems and methods used in many studies. Here, indicate whether each material, system or method listed is relevant to your study. If you are not sure if a list item applies to your research, read the appropriate section before selecting a response.

Materials & experimental systems

n/a	Involved in the study	
☒	☐	Antibodies
☒	☐	Eukaryotic cell lines
☒	☐	Palaeontology and archaeology
☒	☐	Animals and other organisms
☐	☒	Human research participants
☒	☐	Clinical data
☒	☐	Dual use research of concern

Methods

n/a	Involved in the study	
☒	☐	ChIP-seq
☒	☐	Flow cytometry
☒	☐	MRI-based neuroimaging

Human research participants

Policy information about studies involving human research participants

Population characteristics	Our complete population includes nearly half of Israel's population. In our cohort we have 48% male, average age is 59.5 for male, 61.0 for females.
Recruitment	Recruitment was not active as this is a retrospective observational study. Population includes all patients in Clalit healthcare.
Ethics oversight	Clalit Helsinki Committee 0195-17-COM2

Note that full information on the approval of the study protocol must also be provided in the manuscript.
